# The Research Progress of Electrical Impedance Tomography for Lung Monitoring

**DOI:** 10.3389/fbioe.2021.726652

**Published:** 2021-10-01

**Authors:** Yan Shi, ZhiGuo Yang, Fei Xie, Shuai Ren, ShaoFeng Xu

**Affiliations:** ^1^ The School of Automation Science and Electrical Engineering, Beihang University, Beijing, China; ^2^ Department of Pulmonary and Critical Care Medicine, Chinese PLA General Hospital, Beijing, China; ^3^ State Key Laboratory of Fluid Power and Mechatronic Systems, Zhejiang University, Hangzhou, China

**Keywords:** electrical impedance tomography, lung monitoring, functional imaging, application, research progress

## Abstract

Medical imaging can intuitively show people the internal structure, morphological information, and organ functions of the organism, which is one of the most important inspection methods in clinical medical diagnosis. Currently used medical imaging methods can only be applied to some diagnostic occasions after qualitative lesions have been generated, and the general imaging technology is usually accompanied by radiation and other conditions. However, electrical impedance tomography has the advantages of being noninvasive and non-radiative. EIT (Electrical Impedance Tomography) is also widely used in the early diagnosis and treatment of some diseases because of these advantages. At present, EIT is relatively mature and more and more image reconstruction algorithms are used to improve imaging resolution. Hardware technology is also developing rapidly, and the accuracy of data collection and processing is continuously improving. In terms of clinical application, EIT has also been used for pathological treatment of lungs, the brain, and the bladder. In the future, EIT has a good application prospect in the medical field, which can meet the needs of real-time, long-term monitoring and early diagnosis. Aiming at the application of EIT in the treatment of lung pathology, this article reviews the research progress of EIT, image reconstruction algorithms, hardware system design, and clinical applications used in the treatment of lung diseases. Through the research and introduction of several core components of EIT technology, it clarifies the characteristics of EIT system complexity and its solutions, provides research ideas for subsequent research, and once again verifies the broad development prospects of EIT technology in the future.

## Introduction

Medical imaging technology in today’s medical field is already a mature medical technology that can be used to treat various diseases. Current ultrasound, X-ray tomography, magnetic resonance imaging (MRI), and positron emission tomography (PET) have become important detection tools and auxiliary diagnostic methods for clinical disease diagnosis, but these imaging technologies cannot fully meet the needs of clinical disease diagnosis. Most imaging technologies can only detect when organic diseases occur in organs or tissues. This often misses the golden period of treatment of related diseases. At the same time, the traditional imaging technology is usually accompanied by a certain degree of harm to the human body and also cannot be noninvasive and non-radiative. All these put forward higher requirements for new imaging technologies. Electrical impedance tomography (EIT) is a new image reconstruction technology. According to the conductivity of different tissues inside the organism, it can obtain the distribution of conductivity by applying current on the surface of the organism and measuring the voltage, and then realize image reconstruction by image reconstruction algorithm to reflect the internal structure of the biological tissue (A and C, 2018). The conductivity of different biological tissues of human body is usually different, and in different states, the conductivity of the same tissue will show great differences, which is the main working principle of electrical impedance imaging. By measuring human body’s electrical impedance distribution, we can understand some physiological and pathological information of the human body. Compared with the normal electrical impedance distribution, we can use it to diagnose some diseases (Bayford, 2006). Compared with the current commonly used medical imaging equipment X-ray CT, PET, and MRI, its image resolution needs to be improved, but EIT can be used as an auxiliary medical examination and observation method, which can be used to quickly and qualitatively study certain diseases. The dynamic development process of the current medical imaging technology and detection methods cannot be replaced and it has its own unique advantages. The EIT imaging system is easy to operate, simple in structure, and short in data acquisition time. In the face of major sudden infectious diseases, improving the detection efficiency can speed up the isolation of patients and reduce the scope of transmission. At the same time, EIT avoids the use of nuclides or rays that are harmful to the human body. It can be used for multiple measurements over a long period of time, and it can also be used repeatedly. In addition, the electrical impedance imaging equipment is small in size and convenient to carry, which facilitates long-term, bedside continuous monitoring of the patient without causing any damage to the patient.

The core part of the EIT technology is the solution of the forward problem and the image reconstruction of the inverse problem. Its complexity is mainly manifested in the analysis of the numerical method of solving the forward problem and the inverse problem. The problem itself is an ill-posed problem. The “soft field” characteristics and the conductivity distribution inside the imaging body present a nonlinear relationship; in the inverse problem, small disturbances in the electric field data will have a great influence on the final conductivity reconstruction result. In addition, because the equations and known conditions are complex expressions, when the shape of the solution area is complex, we cannot obtain the exact solution through numerical calculations. Therefore, we can only obtain the approximate solution of the equation according to the actual situation. In the process of seeking the approximation, the original equation needs to be discretized, which makes the electrical impedance imaging problem “ill-posed.” In the process of solving the inverse problem, we need to choose the correct image reconstruction algorithm to avoid numerical problems. In addition, the data collected by the hardware system is limited by the accuracy of the hardware system itself and the development of signal processing technology. The measurement result will be affected by skin contact impedance, which affects the final imaging resolution to a certain extent. All of the above characteristics make EIT a very complex system problem. Many researchers have also put forward their own solutions to these complex problems. The main development trend is to continuously improve the resolution of EIT. These specific methods and research will be discussed in detail later.

In this article, the relevant research methods are summarized for the complex core technology parts of the abovementioned EIT technology, and the current EIT-related research progress and clinical application for lung disease are reviewed. The specific article chapters are arranged as follows: in the second chapter, the electrical impedance characteristics and bioelectrical impedance model of biological tissue are briefly described, and the working principle and the commonly used bioelectrical impedance model of electrical impedance imaging technology are explained. The third chapter compares several methods of solving EIT forward problem, classifies several current image reconstruction algorithms of inverse problem from different angles, and summarizes each kind of algorithm; the fourth chapter describes the principle of electrical impedance imaging technology hardware system and describes hardware components of excitation source, electrode, and the data acquisition system. The fifth chapter summarizes some clinical applications of EIT in the lung. This review is comprehensive, covering the core part of the whole electrical impedance imaging technology, and has a certain heuristic research significance.

## Electrical Impedance Characteristics of Biological Tissues

### Biological Electrical Characteristics of Electric Current

It can be seen from the macroscopic view that the human body can be regarded as a mixed conductor with different electrical characteristics, which is distributed in space according to certain rules. The electrical properties of organisms can retain complete pathological and physiological information (Kanti, 2014). The electrical characteristics of organisms will change with different organs and tissues. Generally, the impedance characteristics of different tissues are quite different (Lamlih et al., 2018). Table 1 is electrical conductivity of some common tissues and organs.

**TABLE 1:** Conductivity parameters of human tissues and organs (20–100 KHz).

Biological tissue	Resistivity/ Ω.m	Biological tissue	Resistivity/ Ω.m
Bone	166	Liver	3.5–5.5
Fat	21–28	Blood	1.5
Lung	7.3–24	Plasma	0.66
cerebrospinal fluid	6.8	Nervous tissue	5.8
Gray matter of brain	2.8	White matter of brain	6.8

**FIGURE 1:**
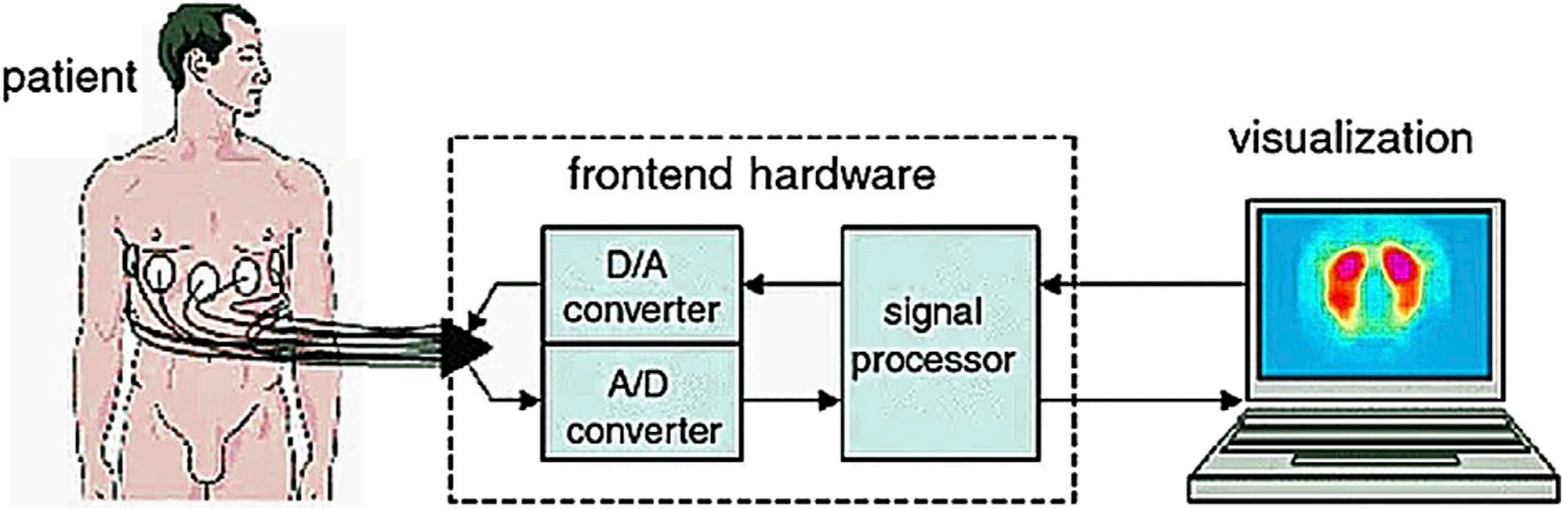
Working process of EIT based on electrical characteristics of biological tissue (Leonhardt and Lachmann, 2012). The data collected from the human body is uploaded to the computer for image reconstruction and the electrical impedance tomography result is obtained.

Through the external input of current signals with different frequencies and amplitudes, the electric field is generated near the measured biological tissue and the resistance distribution around the tissue is obtained. The obtained resistance distribution is finally transmitted to PC through data acquisition and data processing, and an image reconstruction algorithm is used to realize image reconstruction. This is a relatively complete electrical impedance imaging process. It can be seen that the main working principle is the electrical characteristics of biological tissue, as shown in Figure 1 (Leonhardt and Lachmann, 2012).

Through the correlation image reconstruction algorithm, the electrical impedance distribution can be obtained, which can be compared with the standard value to obtain the correlation physiological information of the human body.

In medicine, early diagnosis of diseases can be made according to the change of electrical conductivity, which can be used for detection before the occurrence of substantive lesions and the disease can be detected earlier for preventive treatment. If the frequency of the current injection into the organism remains within the safe current range, it will not cause damage to the organism. We can make use of the electrical characteristics of the organism to conduct electrical impedance imaging and apply it to medical fields such as disease diagnosis.

### Bioelectrical Impedance Model

**FIGURE 2:**
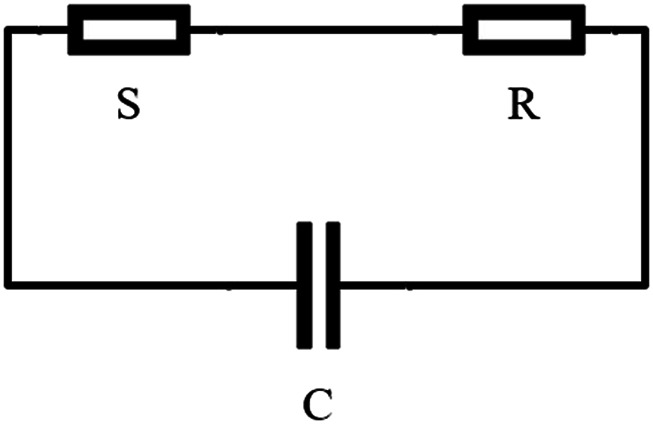
Equivalent circuit of biological tissue. Bioimpedance is equivalent to a three-element circuit composed of only resistance and capacitance.

For the convenience of research, we can characterize the bioelectrical impedance model by some electrical equivalent models (Song-Bin et al., 2015). Among the electrical impedance models, the most basic bioelectrical impedance model is the three element model (Palko et al., 1995; Fraiwan, 2005). The most common three element model (Lu et al., 1996) is that the whole circuit contains the equivalent internal resistance R , equivalent external resistance  S , and equivalent membrane capacitance C . Figure 2 shows the circuit model diagram. Under this model, the bioelectrical impedance can be expressed as Eq. 1, where j is the imaginary part and w is the frequency.

$$Z=\frac{R\left(1+\mathrm{jwcs}\right)}{1+\mathrm{jw}\left(R+S\right)}.$$ (1)

Cole KS proposed the Cole–Cole model in 1941 on the basis of the three element model (Cole and Cole, 1941), which can be expressed as Eq. 2:

$$Z=R_{\infty }\frac{R_{0}-R_{\infty }}{1+{\left(\mathrm{jw\tau}\right)}^{\alpha }},$$ (2)

where Z is the impedance value affected by the frequency. α is the relaxation factor, which controls the capacitance characteristics. $R_{0}$ is the resistance value at low frequency in the initial state and $R_{\infty }$ is the resistance value at high frequency state. On the basis of previous studies, Schwan proposed a new dispersion theory according to the characteristic that tissue electrical impedance parameters have significant changes in a specific frequency band (Schwan, 1957; Schwan, 1983; Schwan, 1993; Schwan, 1994). There are three frequency scattering sections in biological tissue, which are α , β , and γ scattering. In the α frequency scattering region, the change of cell ionic layer leads to the change of cell capacitance. In the  β frequency scattering region, the capacitive short circuit of cell membrane capacitance occurs. Generally, the electrical impedance characteristics of biological tissue studied by us are concentrated in the above two frequency ranges. In the  γ frequency scattering band, mainly high-frequency microwave has serious damage to the human body, so it is not conducive to the study of electrical impedance characteristics of biological tissue.

## Algorithm

### Forward Problem

EIT can be divided into three parts: mathematical modeling, forward problem solving, and inverse problem solving (Lionheart, 2004; Djajaputra, 2005; Liu et al., 2021). Mathematical modeling is to use Maxwell’s equation to establish a mathematical model which can approximately describe the EIT problem. Generally, numerical methods are needed.

The forward problem is to calculate the observed value of the model according to the known model. For EIT, the forward problem is the process of solving the potential distribution or boundary voltage distribution with known excitation current and conductivity distribution. To some extent, the solution of the forward problem is a key step for EIT to successfully complete image reconstruction, because in the calculation of the inverse problem, the result of the forward problem needs to be used for image reconstruction. The traditional methods for solving forward problems are the finite element method (FEM) (Chsner, 2020), the finite element difference method (Abascal et al., 2007), the boundary element method (de Munck et al., 2000), and the finite volume element method (Dong et al., 2005), in which the finite element method is the most widely used (Bagshaw et al., 2003; Lionheart and Paridis, 2010). The main principle of BEM (boundary element method) is to discretize the boundary of the field, divide it into finite elements, and transform the boundary integral equation into algebraic equations. In this way, we can use Gauss theorem to reduce the order, which can help us reduce the difficulty of calculation. However, it is also easy to cause numerical problems. The finite volume element method divides the field to be solved into innumerable main elements, constructs auxiliary elements, calculates the current flux flowing into the control body, and uses this current flux to represent the potential of each node in the main element and further transforms it into a matrix equation, which is solved to obtain the final calculation results. This method can reduce the amount of calculation and improve the accuracy at the same time, but it does not have universal applicability and is restricted by conditions. The solution process of the finite difference method is to use the finite difference grid to discretize the field of the problem to be solved, use the numerical differential function to transform the differential quotient in the definite solution into the difference quotient, and discretize the original problem into the difference scheme to obtain the numerical solution. Similar to the finite volume element method, the finite difference method can also reduce the amount of calculation and simplify the difficulty of calculation, but the accompanying problem is that its application scope needs special boundary requirements, and the efficiency is low in dealing with the curve boundary problem. Therefore, it cannot be widely used in human tissue electrical impedance tomography. The finite element method has become the most commonly used forward problem processing method because it can reduce the amount of calculation and has a wider range of adaptability. The basic principle is to divide the measured object into finite elements, change the partial differential equation into Kφ=I , where K is the coefficient matrix of conductivity of each partition point, φ is the potential of each partition node, and I is the vector set containing various current information, and use the known conductivity distribution to obtain the potential of each partition node by the Gauss elimination method. The only disadvantage of this method is that it does not meet the current continuity conditions of the normal component of current density and the tangential component of the electric field at the interface. The model is too rigid, but in the actual calculation process, this disadvantage will not bring substantial calculation error, so the finite element method is still the most practical method to solve the forward problem.

The above methods can be used to solve EIT forward problems, but the finite element method is the most commonly used method to solve EIT forward problems because of its strong applicability.

### Inverse Problem

Inverse problem refers to the process of calculating the information of the object or the system according to the measurement results. The inverse problem of EIT is to solve the conductivity distribution in the field by using the voltage value obtained from the forward problem and the boundary voltage measurement. The inverse problem is a highly ill conditioned nonlinear problem. Given the measured value V of finite edge voltage and the operator F of the positive problem, we can solve the case which is closest to the real conductivity distribution. Its mathematical expression can be expressed as Eq. 3, where ${\left\|.\right\|}_{2}^{2}$ is L2 norm, σ is conductivity distribution. At present, there are many mature algorithms to solve the inverse problem.

$$E\left(\sigma \right)=\frac{1}{2}{\left\|F\left(\sigma \right)-V\right\|}_{2}^{2}.$$ (3)

The difference algorithm is to make the difference between the boundary voltage values of the same field obtained at two different times under the same excitation and use the difference to reconstruct the image so as to get the change of the impedance distribution in two different fields at different times. The typical difference algorithms include the back projection algorithm (Barber et al., 1983; Santos and Vogelius, 1990), the one-step Newton–Gauss method (Cheney et al., 2010), the sensitivity matrix method (Murai and Kagawa, 1985; Hyeuknam et al., 2013) and so on. Table 2 summarizes the above three differential algorithms.

**TABLE 2:** Several typical difference algorithms.

Method	Principle	Advantage	Shortcoming
Equipotential line back projection	The reconstructed image is obtained by weighted average of multiple calculation results	It can eliminate noise interference and its own error	Only for two-dimensional image
One step Gauss–Newton method	The field conductance distribution is obtained by the least square method	The calculation process is simplified	The initial conductivity requirement must be real
Sensitivity matrix reconstruction algorithm	The relationship between the change of total conduction impedance and the change of single element impedance is established	Eliminates the interference and noise in the signal	The scope of application is small

Since then, most of the difference algorithms are based on the predecessors, adding new ideas. Cao et al. (2019) proposed a new algorithm of time difference electrical impedance tomography based on multi-frequency information. Based on the pseudo measurement principle, an improved nodal back projection algorithm is proposed by Liu et al. (2019). The research shows that the algorithm has significant advantages in accuracy, reliability, and speed (Liu et al., 2019). Tobias et al. (2019) proposed a frequency difference electrical impedance tomography algorithm based on the absolute value, which has strong robustness under noise interference. A new iterative algorithm based on the sensitivity matrix is proposed by Chen et al. (2019).

The absolute algorithm aims at the absolute value of the electrical impedance and directly solves the absolute value of the impedance distribution in the field according to the measured voltage at the boundary of the measured object. Absolute EIT is usually more difficult to implement than differential EIT because it cannot eliminate the error as the differential algorithm. In general, the imaging effect of the absolute electrical impedance imaging algorithm will be affected by the electrode, including electrode motion, electrode contact impedance, and so on. The common way to eliminate the error is to calibrate the electrical impedance imaging system, carefully install the equipment (electrode layout and contact), record the deviation from the planned acquisition, and measure the actual installation position with very high resolution.

In 1987, Yorkey applied the Gauss–Newton method to static imaging, using the product of the first-order partial differential matrix and the Jacobian matrix to approximate the Hessian matrix, which reduces the complexity of the traditional Newton algorithm; in 1991, Silanen et al. proposed the delamination method, which uses the conductivity near the boundary to obtain the boundary data of the inner layer by combining it with the Riccati nonlinear difference equation. After repeated calculation, the conductivity distribution problem of the whole circular object is transformed into the conductivity distribution problem of multiple single layers, which can simplify the calculation difficulty, but there will be instability. On the basis of previous research, Siltanen proposed a D-bar algorithm (Siltanen et al., 2001; Isaacson et al., 2006; Knudsen et al., 2007), which transforms the conductivity problem into a Schrodinger equation and then combines with the sigma method in inverse scattering to solve the problem (Cornean et al., 2006); Mueller and Siltanen (2020) have studied the D-bar algorithm again recently, and summarized various variants and latest development of the D-bar algorithm. Based on the traditional static image reconstruction algorithm, Zhang et al. (2020b) introduced a new proportional genetic algorithm to realize EIT image reconstruction.

At present, several common regularization algorithms can be divided into two categories according to different constraints. The regularization algorithms based on the L-2 norm include the Tikhonov regularization algorithm (Peng et al., 2007) and the noser regularization algorithm; the regularization algorithms based on the L-1 norm include the sparse regularization algorithm (Varanasi et al., 2019) and the TV (Total Variation) regularization algorithm (Fan, 2012). The Tikhonov regularization algorithm is one of the most commonly used regularization algorithms based on L-2 norm. The core idea is to add a penalty function to the objective function to dampen the higher-order eigenvector to obtain the well posed solution. The penalty function is usually a quadratic function. However, this algorithm is only suitable for the image reconstruction of the continuous conductivity target and cannot reconstruct the jumping conductivity target, which limits its scope of application to a certain extent. The NOSER regularization algorithm is also a common regularization algorithm based on the L-2 norm. Its calculation principle is based on the Jacobian matrix. Generally, the prior information added is a rule: the more sensitive the cell is, the higher the contribution value to the boundary voltage is. In other words, among all the cells, the sensitive cells play a decisive role in boundary voltage. This method improves the accuracy by punishing the cells with high sensitivity. At present, the main problem is that the resolution is low when facing the conductivity target with uneven distribution, and it needs to be further improved. The sparse regularization algorithm is a regularization algorithm based on the L-1 norm. Similar to the regularization algorithm mentioned above, the core idea is to add the L-1 norm of the solution to the problem as a penalty function and use the L-1 norm to obtain some details and reduce the difficulty of calculation. It can improve the shortcoming of the Tikhonov regularization algorithm which is too sensitive to the boundary, and it can also solve some discontinuous problems when the boundary changes dramatically. The TV regularization algorithm is also a constraint term based on the L-1 function. Different from the previous methods, it uses the discrete variogram of an EIT conductivity reconstruction image as its constraint term, which can enhance the discontinuity of imaging, retain the discontinuous changes in the reconstruction results, and meet the requirements of the scale. In the case of a continuous objective function, it cannot achieve good results because of the staircase effect. In the actual use process, we should try to reduce the impact of the staircase effect to obtain higher resolution imaging results.

The subsequent research on regularization algorithm is mostly based on the two research, Borsic et al. (2009) tried to solve the time division inverse problem of TV regularization by using the primal dual interior point method; Yoon et al. (2014) proposed a first-order TV regularization algorithm—linear alternative direction multiplier algorithm, which was used to solve the EIT inverse problem; Liu et al. (2013) also combined the total variation regularization algorithm with the Tikhonov regularization algorithm to obtain better reconstructed images; Lysaker et al. (2003) proposed using the second-order differential to construct the regular LLT (Low-Latency Trend line) model and obtained better practical results, but it is easy to produce fuzzy phenomenon in the image edge; in 2019, Shi et al. (2019) proposed a regularized ladder effect suppression algorithm based on generalized variation, which can meet the needs of high resolution, suppress the ladder effect, and improve accuracy; Ahmad et al. (2019) used statistical inversion method based on Bayes theorem and an iterative regularized Gauss–Newton method for image reconstruction of EIT; Wang (2020) proposed a non-convex p-norm sparsity-promoting regularization. Compared with L-1 norm regularization, it improves the spatial resolution and is more robust to noise.

An intelligent reconstruction algorithm is also a commonly used algorithm to solve EIT inverse problem. The common intelligent reconstruction algorithms include a Hopfield network method, a steepest descent BP (Backpropagation) learning method, and a deep learning method. The Hopfield network method solves the conductivity distribution by establishing the energy function of EIT imaging based on the Hopfield network; the steepest descent BP learning method solves the conductivity distribution by learning the mapping relationship between boundary measurement voltage and conductivity (Zhang et al., 2020a). By using the deep structure and self-learning ability of the network, the mapping relationship between the boundary voltage and the conductivity is learned and the conductivity distribution is obtained. Some new EIT image reconstruction algorithms are emerging in recent years. Li et al. (2020) designed an image reconstruction algorithm based on an one-dimensional convolutional neural network; Huang et al. (2019) used the idea of artificial neural network to improve the resolution of reconstructed image; Barbosa et al. (2019) summarized the application of the fish swarm algorithm, non-blind search, and a genetic algorithm in solving EIT inverse problem; Liu et al. (2020) proposed an electrical impedance tomography time sequence learning based on Bayesian space-time prior; Mendoza et al. (2020) studied the effect of six heuristic algorithms applied to EIT image reconstruction. Li et al. (2019) proposed a new deep neural network algorithm for image reconstruction. Husain and Liatsis (2019) proposed a local decomposition image reconstruction algorithm based on a neural network.

## Hardware

**FIGURE 3:**
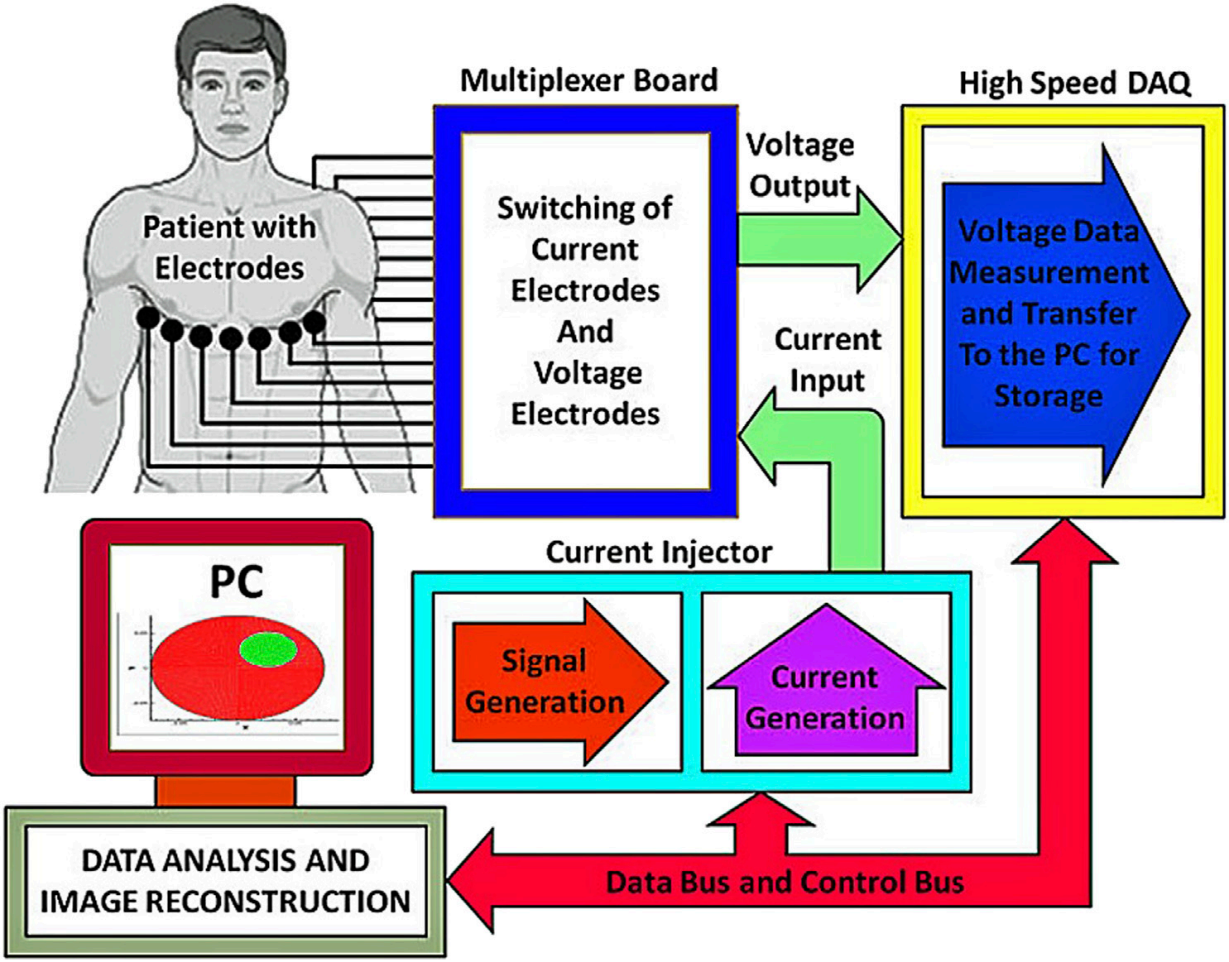
Schematic of an EIT instrumentation system [adapted from Bera and Jampana (2011)]. The main structure of the hardware system is composed of an electrode array, an excitation source, a data acquisition system, an upper computer, and a strobe switch.

The hardware system is a key component to realize EIT. The accuracy of the signal collected by the hardware system directly affects the resolution of the image obtained by the final image reconstruction. The continuous improvement of hardware system technology provides EIT with higher-precision processing data, which helps to solve the shortcomings of low resolution of EIT imaging results. The EIT system consists of an electrode array, data acquisition, and image reconstruction (Mohamedali et al., 2019). The whole electrical impedance imaging system includes digital controller, analog-to-digital (A/D) and digital-to-analog (D/A), current source, a differential voltage sensor, a switching mechanism between electrodes, wiring between the system and electrodes, and electrodes itself. The image reconstruction part mainly depends on the algorithm in Chapter 3. An electrode array mainly includes the number, material, and shape of electrodes. Signal acquisition consists of an excitation channel, a measurement channel, a multiplexer, and a communication module. After the completion of data acquisition, the data is transmitted to the computer through the serial port, and the electrical impedance image reconstruction is realized by using related algorithms. Figure 3[adapted from Bera and Jampana (2011)] shows the schematic diagram of the EIT system. Next, we will introduce several parts of the hardware system.

### Excitation Device

The signal source is an important part of the EIT hardware system. A signal generator is a common signal source at present. In order to meet the needs of signal generator in many fields, the output signal of frequency synthesis technology needs higher output precision and larger output frequency range. At present, a direct digital frequency synthesis (DDS) is a common frequency synthesis technology. In 1971, Tierney et al. (1971) proposed direct digital frequency synthesis technology. Since then, the DDS technology has been developed continuously. The current DDS signal generator has high-frequency resolution, a wide spectrum range of the output signal, low noise of the output signal, and can generate an arbitrary waveform signal according to the actual needs. The excitation device in EIT can also use the DDS signal generator.

In the EIT hardware system, the excitation device is needed to generate a sinusoidal signal. We can use the special DDS (Direct Digital Synthesizer) chip and the internal integrated phase accumulator module to determine the output signal frequency with the input frequency control word. This method is relatively fixed, and it is easy to be limited by the fixed parameters when using. In addition, we can also use FPGA (field programmable gate array)/CPLD (complex programmable logic device) to replace the function of a phase accumulator module in DDS principle (Artem and Dmitry, 2013) and then convert the digital output into an analog signal through external DAC (digital to analog converter) (Zarafshani et al., 2010; Baidillah et al., 2019). A typical digital controller is composed of FPGA and a microcontroller. The microcontroller cooperatively performs calculation, stores data, and transmits the results to the host system. FPGA controls the current source and the measurement function through A/D and D/A converters, drives current, and measures voltage. This method can adjust the number of bits of phase accumulator according to the need of internal design and greatly reduce the design cost. It not only has higher resolution and shorter conversion time but also has higher integration. It is the most suitable signal source excitation device at present.

### Electrode

An electrode is an important component of the EIT hardware system. It directly participates in the process of signal excitation and measurement. When selecting electrode materials, we should fully consider its conductivity, contact area, stability, and cost. At present, in order to improve the durability of the electrode, titanium alloy has been used as the electrode manufacturing material (Salama et al., 2017). In addition, the shape of the electrode can affect the uniformity of the current density distribution in the measured field. At present, the more common electrodes are: a circular electrode, a rectangular electrode, and a composite electrode (Sakamoto and Kanai, 2020). The EIT system often uses a single electrode and a composite electrode structure (Boyle and Adler, 2011).

**FIGURE 4:**
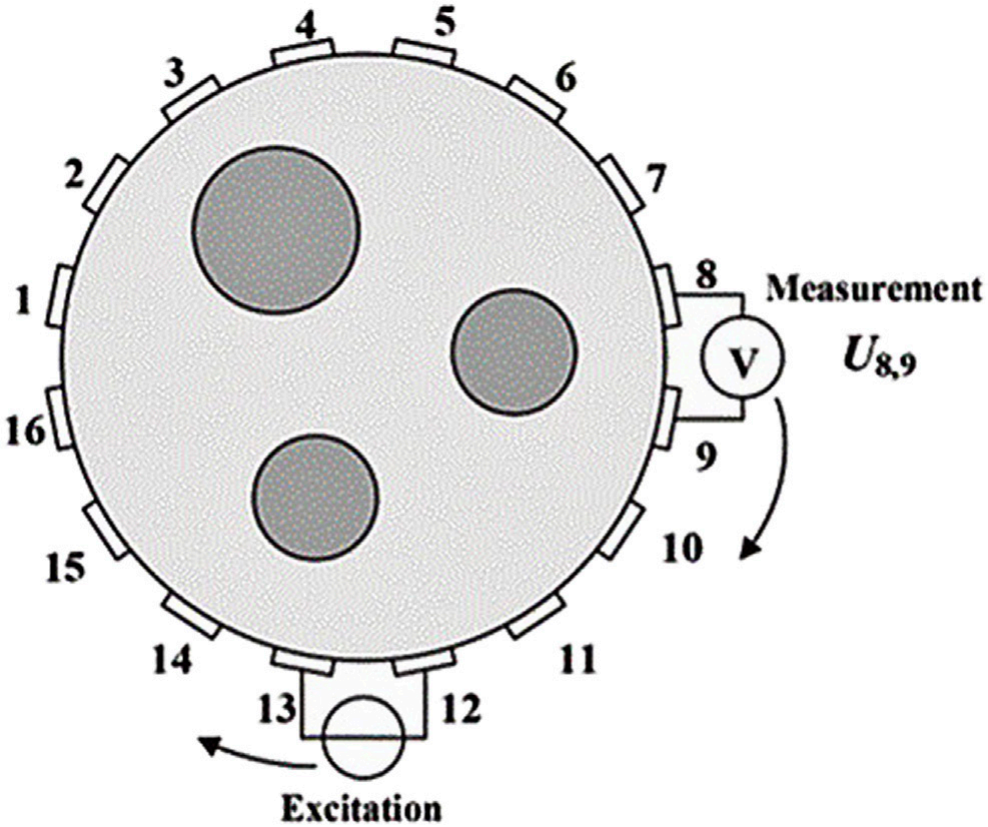
Working principle of a 16 electrode system (Wang et al., 2020). Adjacent excitation is to select a pair of adjacent electrodes to input safe current, and then measure the output voltage between several pairs of adjacent electrodes except the excitation source.

The number of electrodes directly determines the number of measurement signals that can be obtained. When the number of electrodes is too large, it can collect as many signals as possible for the imaging quality. However, the excessive number of electrodes will also increase the measurement time of the whole system, increase the difficulty of calculation, and slow the imaging speed. Therefore, it is necessary to select the number of electrodes according to the actual situation. At present, the most common EIT hardware systems are a 16 electrodes measurement system, a 32 electrodes measurement system, and a 128 electrodes measurement system. Figure 4 (Wang et al., 2020) shows the working principle of the 16 electrodes system.

The electrode position is also a factor affecting the imaging quality. The excitation electrodes were placed between the ribs of axillary midline on both sides of thoracic cavity, and the two measuring electrodes were located inside the two excitation electrodes.

**FIGURE 5:**
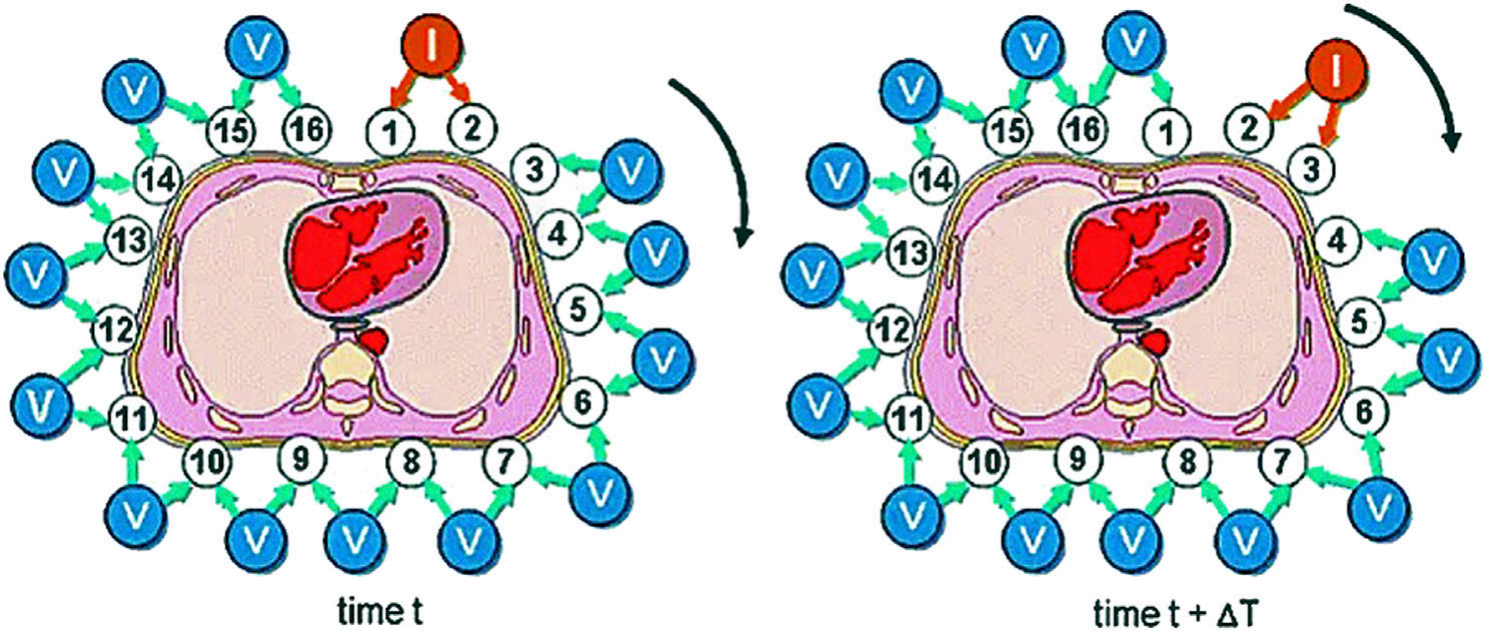
Working process of the adjacent driving mode (Leonhardt and Lachmann, 2012). After the selected set of excitation tests are completed, we replaced the excitation source electrodes in a clockwise direction and continue to measure the output voltage.

The existing driving modes can be roughly divided into an adjacent driving mode (Zong et al., 2020), a relative driving mode, and an inductive driving mode (Ribeiro et al., 2021). Adjacent drive is to select two adjacent electrodes as the current excitation input and measure the voltage between the two adjacent electrodes except the two electrodes in turn, which is easier to implement in hardware with high quality. Relative drive is to select two relative electrodes as the current excitation input and measure the voltage between the two adjacent electrodes except the two electrodes in turn. The data obtained is less than the adjacent driving mode. The time-varying current is applied to induction coil to generate induced current, and the output voltage is measured through the surrounding electrodes, so this way is still in further development. In practical application, the adjacent incentive mode is still used most. Figure 5 (Leonhardt and Lachmann, 2012) shows the working process of the adjacent driving mode. After the selected set of excitation tests are completed, they replaced the excitation source electrodes in a clockwise direction and continue to measure the output voltage.

### Data Acquisition System

A data acquisition system is the core part of the hardware system, and it is the main link to overcome the influence of error and noise (Shi et al., 2018). The EIT data acquisition system includes a main control module, an excitation module, a signal detection module, a data acquisition module, a drive measurement control module, and a computer interface module.

The main control module of the system controls the excitation module to generate the excitation signal with certain amplitude and frequency, and makes the excitation signal act on the measurement target through the excitation electrode selected by the drive measurement module. At the same time, the main control module controls the signal detection module, the data acquisition module, and the drive measurement control module to pick up the boundary voltage of the target surface and transmit it to the computer through the interface.

In EIT hardware system measurement, phase sensitive demodulation technology is needed to process the measured signal, and the real and imaginary parts of the measured signal can be obtained. At present, the commonly used phase sensitive demodulation technology can be divided into analog demodulation and digital demodulation. Analog demodulation mainly includes analog multiplier demodulation, switch demodulation, sample and hold demodulation, and zero crossing phase demodulation (Sakamoto and Kanai, 2020). Digital demodulation technology mainly includes Fourier transform demodulation technology. For analog multiplier demodulation, the principle is to input an analog reference signal with the same frequency and phase and multiply the measured signal and the reference signal in the four quadrant multiplier. For switch demodulator, it takes the square wave as the reference signal and has stronger anti-interference ability, but the imaging speed is slow. For a positive signal, the switch device outputs “+1”; for a negative signal, the switch device outputs “-1.” For sample and hold demodulation, it stores the value, keep it in a certain time range, and then transfer the value to the analog-to-digital converter for conversion to get the value of the input signal at a specific time point. For zero crossing phase demodulation, two independent circuits are used to measure the RMS and phase information of the alternating signal. After the two input signals are processed by filter and shaping circuit, the signal is sent to the phase detector, and the phase discriminator will output the pulse signal. For Fourier transform demodulation, it is implemented by FPGA, which can be flexibly adjusted according to the frequency, which has high demodulation speed and difficult to control the frequency synchronization of the demodulated signal and the sampled signal.

At present, with the maturity of theoretical knowledge and the development of hardware technology, many complete EIT devices have been successfully produced, which are composed of the main components of the above parts of hardware. Table 3 [adapted from Putensen et al. (2019)] shows the core hardware components of the EIT devices produced by several major companies.

**TABLE 3:** Hardware composition of EIT in some production companies [Adapted from Putensen et al. (2019)].

Company	EIT system	Electrodes		Measurement and data acquisition
Swisstom AG	BB2	32	Electrode belt	Pair drive serial measurement
Timpel SA	Enlight	32	Electrode stripes	Pair drive parallel measurement
CareFusion	Goe-MF	16	Individual electrodes	pair drive serial measurement
Dräger Medical	PulmoVista 500	16	Electrode belt	Pair drive serial measurement
Maltron Inc.	Mark 1	16	Individual electrodes	Pair drive serial measurement
	Mark 3.5	8	Individual electrodes	Pair drive serial measurement

### Research Status of EIT Equipment

**FIGURE 6:**
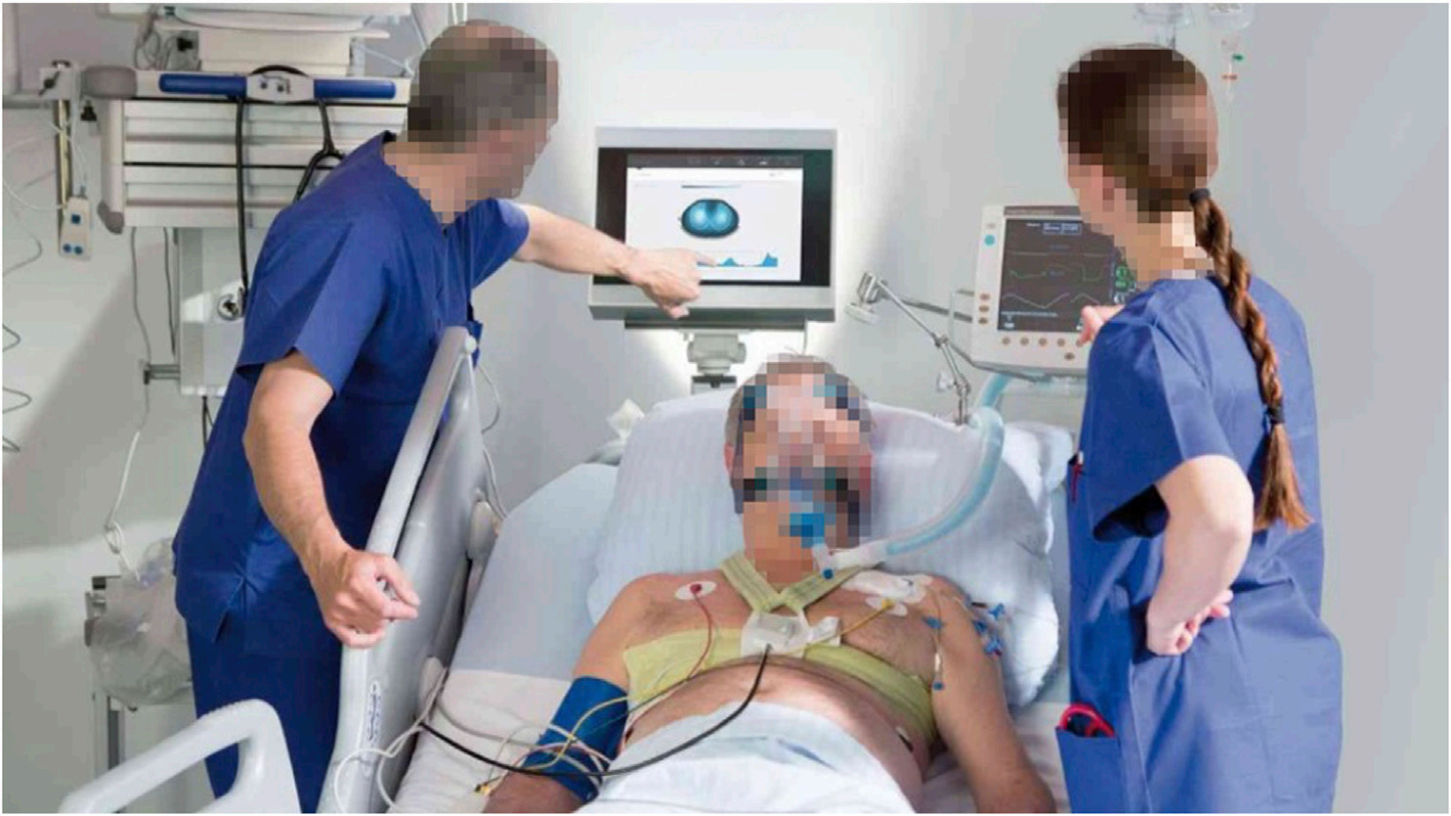
The EIT device BB2 of Swisstom. Swisstom’s equipment is used in the clinical treatment of patients with acute respiratory distress.

**FIGURE 7:**
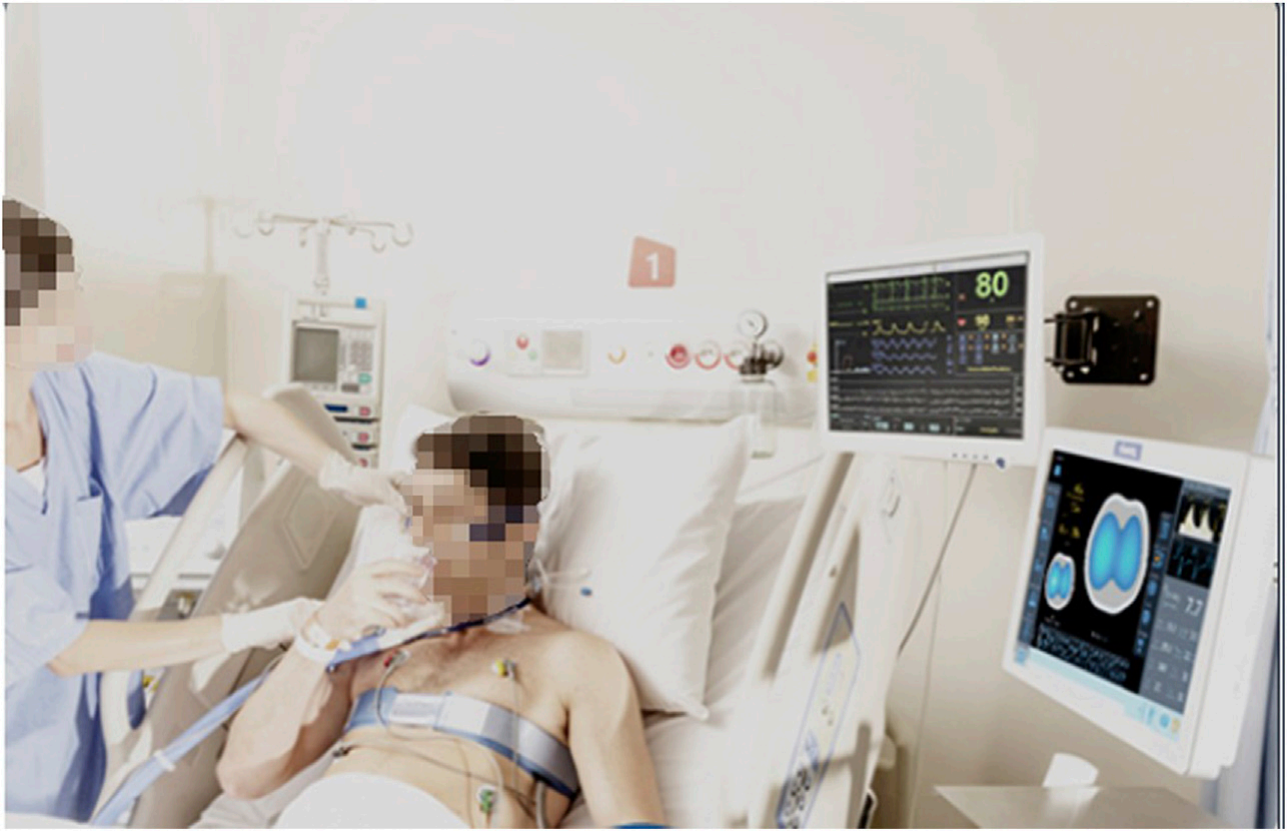
The EIT device Enlight 1800 of Timper. Timper’s Enlight 1800 can monitor the patient’s lungs in real time, complete functional imaging and lung ventilation monitoring.

In 2005, researchers at Tel Aviv University in Israel designed an 8-electrode portable bioelectrical impedance monitoring system for detecting changes in lung resistivity (Zlochiver et al., 2007). By detecting the changes of left and right lung resistivity, the system can help doctors control drug use according to the patient’s condition. In 2007, Kyung Hee University and University College London designed a set of digital multi frequency EIT system. The system uses DSP as the main control processor and FPGA as the coprocessor. It uses digital technology to complete the analog filtering and analog multiplication demodulation in the traditional EIT system, which improves the working speed and reduces the noise. In 2008, researchers from Israel’s Hebrew University and the University of California, Berkeley, designed a 32 electrode portable EIT system (Yair et al., 2008). The system can transmit the voltage measurement results of the electrode to the mobile phone and then connect to the central computer through the mobile phone dial-up to reconstruct the image. Finally, the computer can send the reconstructed image back to the mobile phone for patients to view or doctors to diagnose. The school of information technology of Beijing University of Chemical Technology uses DSP as the main controller to measure the multi-frequency high-resolution bioimpedance. The first generation of electrical impedance breast detector developed by Biomedical Engineering Department of the Fourth Military Medical University has passed the national medical device registration and entered the research of clinical application. At present, the second generation of portable electrical impedance detection system is being studied. Zamora-Arellano et al. (2020) designed a portable, reliable, and low-cost EIT image reconstruction system based on the embedded system. The PulmoVista 500 developed by Dräger has successfully applied EIT technology to the clinic. It can provide visual patient ventilation monitoring. It has many advantages such as noninvasive monitoring, non-radiation, repeatable, real-time continuous, fast response, and bedside measurement. However, this imaging technology uses the contact electrode mode to measure the body surface potential, and the amount of measurement information obtained is small and the spatial resolution is low. It is necessary to confirm the location of the lesion area with the help of traditional imaging techniques such as CT (Computed Tomography) and the measurement accuracy will be affected by many interference factors. Such as the humidity and roughness of the skin surface, the pressure applied during the measurement, and the low stability and sensitivity. In addition, some other medical device manufacturing companies, such as Swisstom, Timper, and other companies, have launched various commercial electrical impedance tomography systems, and the related clinical research has gradually become a research hotspot. Figure 6 is an EIT device BB2 used by Swisstom to monitor lung ventilation. Figure 7 shows the EIT equipment Enlight 1800 designed and produced by Timper, which has been applied to the treatment process of clinical cases. Some specific hardware components of the abovementioned devices are listed in detail in Table 3.

## Clinical Application

The physiological and pathological information of the human body can be expressed through the electrical impedance characteristics of biological tissues. Although compared with the traditional imaging technology, electrical impedance imaging technology still has the disadvantage of poor resolution, but because of its noninvasive, non-radiation, functional imaging, and other advantages, electrical impedance imaging technology is still developing in recent decades, and has been widely used in biomedical engineering and clinical medicine (Walsh and Smallwood, 2016; Frerichs and Zhao, 2019). Because the lung is close to the body surface, the changes of bioimpedance in the chest can be easily monitored (Gong et al., 2015). Therefore, the most widely used in clinical application is some applications in lung pathology (Cherepenin et al., 2002; Lobo et al., 2018). The following is a brief introduction of several EIT in the treatment of lung disease in the practical application.

### Pulmonary Ventilation Monitoring

**FIGURE 8:**
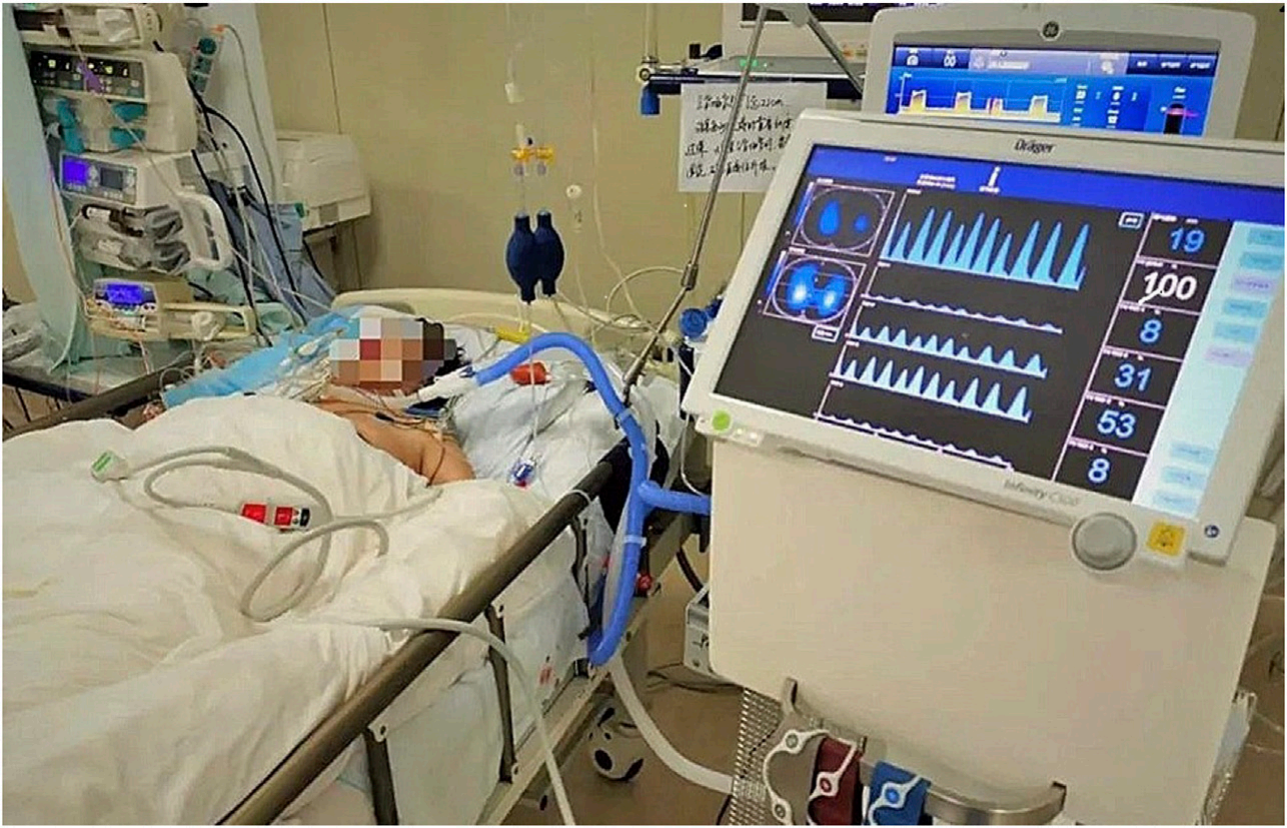
Real-time monitoring of lung disease patients by EIT. Through the EIT equipment, in some clinical pathological treatments, the patient’s pulmonary function imaging and the patient’s long-term bedside continuous monitoring can be achieved.

Protective ventilation requires real-time monitoring of regional lung ventilation to determine the distribution of lung ventilation such as hyperventilation, repeated opening and closing, alveolar collapse, and other factors that can cause uneven lung phenomena, and it is an indicator of insufficient lung ventilation and metabolism. On the basis of the assessment, we can set the ventilation parameters of the lung area with physiological diversity in a targeted manner. In addition, through real-time monitoring of the lung function of patients and accurate prediction of their recovery, it is expected to reduce the patient’s dependence on the ventilator, and at the same time, it can also avoid the risk of premature withdrawal of the ventilator. In 1987, Brown et al. (1985) was the first to use EIT to monitor the changes of human lung impedance. In 1996, Hahn et al. (1996) put forward the concept of lung functional imaging with EIT. In 2003, Hinz et al. (2003) studied the linear relationship between lung impedance change and inspiratory volume and proved that there was a great correlation between them. Pulmonary ventilation monitoring is an important strategy for pulmonary pathological treatment. In 1978, Henderson and Webster (1978) began to use EIT to monitor lung ventilation, which was used to reconstruct the cross-sectional image. In the later clinical application, the concept of regional impedance waveform is introduced. Compared with the global impedance waveform, the regional impedance waveform shows the sum of impedance changes in a specific ROI (region of interest), which can compare the impedance changes in different regions of the lung, so as to compare the regional ventilation distribution of the lung. It also can realize the monitoring of lung diseases and can be applied in the treatment of severe respiratory diseases. Figure 8 shows the clinical application of EIT equipment to realize pulmonary ventilation monitoring function.

### Respiratory Distress Syndrome in Critically Ill Patients With ARDS (Acute Respiratory Distress Syndrome)

For most people with ARDS, mechanical ventilation is a mature method to maintain normal breathing of patients, but unreasonable ventilator parameters may aggravate the lung condition and even cause serious lung injury (Prost et al., 2011). Therefore, the most common protective ventilation strategy is to reduce lung injury by setting a reasonable PEEP (positive end-expiratory pressure) value (Guerin, 2011; Brabant et al., 2019). As a noninvasive medical imaging technology, EIT can monitor the air distribution of mechanical ventilation in real time by imaging the cross section of thoracic cavity (Blankman and Gommers, 2012; Andiani et al., 2019). At the same time, EIT can distinguish the area of collapse and transitional expansion (Bikker et al., 2010; Bikker et al., 2011). For the setting of the PEEP value, Zhao et al. (2010) proposed the global heterogeneity index to determine the optimal PEEP value. Costa et al. (2009) defined the local impedance compliance parameters and defined the areas with compliance values lower or higher than the optimal values as lung collapse areas and over expansion areas. At present, a number of studies use EIT to accurately measure the distribution of the whole lung and regional lung ventilation and realize a personalized protective ventilation strategy by titrating the best PEEP with EIT (Hsu et al., 2016; Guillaume et al., 2017; Heines et al., 2019).

### Pulmonary Perfusion

Similar to lung ventilation, blood flow usually brings changes in some tissue components in the body. Because the impedance changes brought by the changes in components can be captured by electrical impedance imaging technology for monitoring, EIT is also widely used in monitoring blood perfusion. At present, there are two mature methods to monitor lung perfusion: one is to separate the heart beat signal, the commonly used image processing methods are a ECG (Electrocardiogram)-gated acquisition method, a frequency domain filtering method, and a principal component analysis method (Nguyen et al., 2012), and the other is to evaluate blood perfusion by injecting a large concentration of NaCl solution as a contrast agent. Compared with the former, the monitoring effect of injecting larger concentration of NaCl solution is better. McArdle et al. (1988) and Brown and Barber (1994) found that EIT can monitor the damaged perfusion area of the lung, and confirmed that EIT is helpful in the diagnosis of PE (Pulmonary Embolism) through simulation study. Fagerberg et al. (2010) studied EIT of acute lung injury and found that EIT can be used to evaluate the heterogeneity of regional ventilation. For the diagnosis of PAH (Pulmonary Artery Hypertension), Smith et al. Performed EIT in 21 patients with idiopathic PAH and 30 healthy controls. Compared with the control group, EIT showed a low perfusion impedance change signal. This study shows that EIT can be used as a noninvasive technique in the diagnosis and treatment of PE/PAH (Smit et al., 2006).

### Neonatal Pulmonary Function Imaging

**FIGURE 9:**
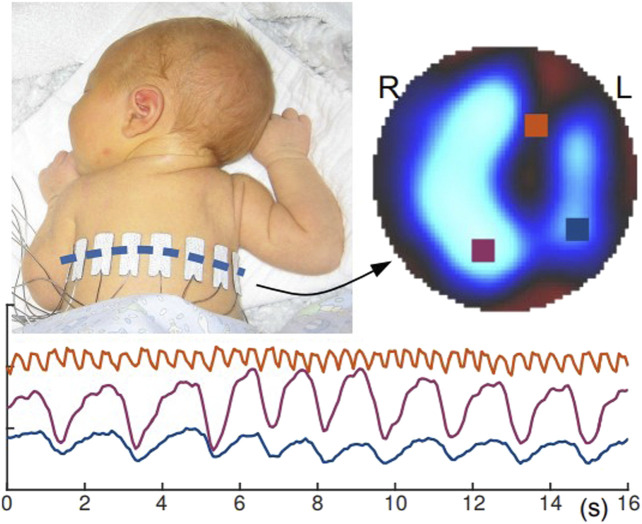
A 10-day-old infant with EIT electrodes (Heinrich et al., 2006). By performing lung function imaging of newborns, timely diagnosis and treatment of lung diseases in early development of newborns without radiation damage can be done.

In the neonatal population, millions of infants are born prematurely every year, and many suffer from respiratory distress syndrome (RDS) due to immature lungs (Chatziioannidis et al., 2008; Durlak and Kwinta, 2013). Although the current mechanical ventilation method can improve the survival rate of patients, it can also cause serious damage to the fragile lung, making the chronic lung disease to continue in adult (King et al., 2019). The incomplete development of the newborn’s own organs and the lack of exposure to radiation detection devices all put forward higher requirements for the imaging technology of newborn children’s lung function. We need a non-radiation, noninvasive imaging technology to image children’s lung function (Durlak and Kwinta, 2013). This device not only needs to meet the needs of pathological knowledge detection and diagnosis but also needs to minimize its impact on neonatal organ development, so some traditional functional imaging will cause varying degrees of damage to the neonatal lung. Therefore, EIT can provide noninvasive and non-radiation detection method for the diagnosis of children’s respiratory and circulatory diseases, which has become a new method for monitoring early neonatal lung disease. Figure 9(Heinrich et al., 2006) shows the lung function imaging of a 10 day newborn in EIT equipment.

In conclusion, because of its unique advantages, EIT has been widely used in biomedical engineering, especially in the pathological research of lung. EIT is often used in the treatment of some lung failure diseases (Frerichs and Zhao, 2019). In addition to the application in lung diseases, there are also some clinical applications in tumor detection or early detection of some diseases for EIT (Ma et al., 2014; Dai et al., 2018; Lee et al., 2020; Yang et al., 2020). With the continuous development of EIT theory and technology, the clinical application of EIT technology will be more and more.

## Conclusion

The development of electrical impedance imaging technology has become more and more mature. The improvement of the hardware system and the image reconstruction algorithm provides the basis for the development of electrical impedance imaging technology. At present, electrical impedance imaging technology has begun its clinical application and has been applied to lung pathology, tumor detection, real-time monitoring, and other medical fields, constantly improving the image resolution and measurement accuracy in practice; its unique advantages can also prove that it has a bright application prospect in the medical field. The application of EIT in clinic also proves that it can play a unique role in some special medical occasions. Its functional imaging can well solve the problem that many diseases cannot be diagnosed early. Moreover, with the maturity of some image reconstruction algorithms and the continuous improvement of hardware system accuracy, its low resolution shortcomings will be gradually made up, and it can also be used in clinical practice. In this study, through a comprehensive summary of all aspects of EIT technology, we have a deep understanding of EIT technology. In this study, the current common methods and principles of each part are described in detail and each part introduces the main working principle and the research results achieved so far. Through this review, we understand the current research status and future development trend of related technology fields at home and abroad, which can provide ideas for the follow-up EIT-related technology research.

At present, the biggest problem of EIT technology is still the transformation from the theoretical part to the practical clinical application. The latest theoretical achievements are constantly developing, but only a part of the function is realized in the final clinical application. Therefore, the future development of EIT technology will be more inclined to transform the theory into practical application (Bayford and Polydorides, 2019). For the image reconstruction algorithm, the main research direction in the future is to continue to study the related image reconstruction algorithm to reduce the error interference. For the hardware system, the main development trend in the future is to continue to improve the accuracy of the hardware system and the accuracy of the measured data, so as to improve the resolution of the final imaging results through the image reconstruction algorithm. In short, EIT-related technologies will continue to develop in the future and play an irreplaceable role in practical medical applications.
